# Methicillin-resistant *Staphylococcus aureus* nasal colonization in a department of pediatrics: a cross-sectional study

**DOI:** 10.1186/1824-7288-40-3

**Published:** 2014-01-10

**Authors:** Francesco Gesualdo, Manuela Onori, Dafne Bongiorno, Floriana Campanile, Emanuela Carloni, Livia Mancinelli, Cristina Russo, Alberto Villani, Diletta Valentini, Massimiliano Raponi, Alberto E Tozzi, Stefania Stefani

**Affiliations:** 1 Bambino Gesù Children’s Hospital IRCCS, Rome, Italy; 2 Department of Bio-Medical Sciences, Section of Microbiology, University of Catania, Catania, Italy

**Keywords:** Methicillin-resistant *Staphylococcus aureus*, Nasal carriage, Risk factors

## Abstract

**Background:**

We describe methicillin-resistant *Staphylococcus aureus* (MRSA) nasal carriage at admission in patients admitted to a Department of Pediatrics.

**Methods:**

All patients received a nasal swab at admission. A questionnaire was administered and molecular genetics analyses were performed on all identified MRSA isolates.

**Results:**

We enrolled 785 patients, affected with both acute and chronic diseases. MRSA nasal colonization prevalence was 1.15% (CI: 0.5607%-2.093%). Methicillin-sensitive *Staphylococcus aureus* (MSSA) nasal colonization prevalence at admission was 19.75% (CI 17.07%-22.64%). Only one MRSA isolate carried the SCC*mec* V variant; all other isolates carried the SCC*mec*IV variant. Five out of 9 MRSA-colonized patients had an underlying condition. Antibiotic therapy in the previous 6 months was a protective factor for both MRSA (OR 0,66; 95% CI: 0,46-0,96) and MSSA (OR 0,65; 95% CI: 0,45-0,97) colonization. A tendency to statistical significance was seen in the association between hospitalization in the 6 months prior to admission and MRSA colonization at admission (OR 4,92; 95% CI: 0,97-24,83). No patient was diagnosed with an *S. aureus* infection during hospitalization.

**Conclusions:**

The majority of our MRSA colonizing isolates have community origins. Nevertheless, most MRSA-colonized patients had been hospitalized previously, suggesting that strains that circulate in the community also circulate in hospital settings. Further studies should elucidate the role of children with frequent contact with health care institutions in the circulation of antibiotic resistant strains between the hospital and the community.

## Background

In the last decade, after an increasing trend in the incidence of methicillin-resistant *Staphylococcus aureus* (MRSA) infections in Europe and the United States, both in adults and in children [1,2], encouraging data on a general reduction of invasive MRSA infections have been recently reported [3,4]. The proportion of *S. aureus* isolates identified as methicillin-resistant is stabilizing or decreasing in most European countries [5]. Nevertheless, MRSA remains a public health priority as the proportion of MRSA of all *S. aureus* isolates is still above 25% in 8 out of 28 countries, mainly in Southern and Eastern Europe, including Italy [5].

Reports on MRSA nasal colonization in children have shown variable prevalence figures, ranging from 0 to over 20%, with European figures of 3.3% or lower [6]. Most of the surveys that investigated nasal carriage have been conducted on healthy pediatric populations, or on patients admitted to Intensive Care Units [7]. Only few studies have investigated nasal carriage in General Pediatric Wards [8].

MRSA nasal carriers may be at greater risk of developing an MRSA infection compared to methicillin-sensitive *S. aureus* (MSSA) carriers and to non-carriers, in particular in intensive care unit (ICU) settings [9,10]. Therefore, programs for preventing MRSA nosocomial transmission have been implemented, including screening of high-risk patients, isolation of colonized individuals and decolonization [11,12]. However, a comprehensive evaluation of prevention strategies is still lacking, and some strategies may have an unexpected impact on colonized children and their families, possibly leading to a feeling of social stigma [13].

The objectives of the present study were to assess the prevalence of MSSA and MRSA nasal colonization among patients admitted to a pediatric department, to describe molecular genetic characteristics of the colonizing isolates, and to identify epidemiological risk factors associated with MSSA and MRSA nasal colonization.

## Methods

### Setting

The Bambino Gesù Children’s Hospital, located in Rome, Italy, is a 607-bed comprehensive medical centre for pediatric healthcare and research. The Department of Pediatrics includes 132 beds in one single building divided into 4 floors by level of assistance.

### Study design and patient recruitment

Over a 3-month period between February 8, 2010 and May 14, 2010 we conducted a cross-sectional study enrolling patients admitted to 3 wards belonging to the Department of Pediatrics. Children admitted to the wards were affected by general pediatric acute or chronic illnesses, rheumatologic diseases, infectious diseases (excluding HIV), bronchopneumopathies, cystic fibrosis, endocrinological diseases and genetic diseases.

No age or pathology constraints were applied in the selection of the population. Patients not speaking Italian, English, Spanish and French were excluded. Written, informed consent was obtained from patients older than 18 years of age, or from parents of patients younger than 18 years of age, together with patient assent, or written, informed consent if patient was older than 12 years.

Enrolled patients underwent an anterior nasal swab in one of the nares within 12 h of admission. A questionnaire was administered to the parents or to the patient to collect demographic and epidemiological data, including: birth date, sex, parents’ data (age, level of education and employment), number of siblings, number of household members, number of bedrooms, presence of smoking people at home, presence of domestic animals, presence of an household member working in a healthcare facility, current breastfeeding (for children < 2 years), daycare center or school attendance, extra-school sport activity (for children > 4 years), nail biting (for children > 4 years), diagnosis of a chronic underlying disease, past diagnosis of pneumonia or of an invasive infection, emergency room visit during last 6 months, hospital admission during last 6 months, administration of antibiotic therapy during last 6 months, upper respiratory tract infection during last week. The study was approved by the Bambino Gesù Children’s Hospital Ethical Committee.

### Laboratory methods for *S. aureus* identification

Samples were collected from anterior nares using the ESwab (Copan, Brescia, Italy) collection and transport device, composed of a flocked swab adsorbed into 1.0 ml of Amies liquid transport medium. For each nasal sample two aliquots (10 μl each) of ESwab liquid medium were streaked onto mannitol salt agar, Chapman (bioMérieux, Marcy l’Etoile, France) and on chromogenic agar MRSA*Select* (Bio-Rad, Nazareth Eke, Belgium) respectively, and incubated at 37°C for 18–24 h. Colonies grown on Chapman agar having morphology suggestive of *S. aureus*, or mauve in color presumptively MRSA positive on chromogenic agar were subcultured onto 5% sheep blood agar. Phenotypical identification was assessed by Vitek2 system (bioMérieux Marcy l’Etoile, France) according to the manufacturer’s instructions.

*In vitro* susceptibility testing for oxacillin, erythromycin, clindamycin, gentamicin, ciprofloxacin, rifampicin, trimethoprim-sulfamethoxazole, mupirocin, vancomycin, teicoplanin (Sigma Chemical, St. Louis, MO, USA) and linezolid (Pfizer, Groton, CT, USA), was further performed by the broth microdilution method to determine the minimum inhibitory concentrations (MICs), following the CLSI guidelines. The EUCAST guidelines were also used for comparison [14,15]. In particular the oxacillin susceptibility was interpreted according to CLSI recommendations, and in cases of discrepancy susceptibility testing was repeated.

The penicillin-binding protein (PBP2a) latex agglutination test (SLIDEX MRSA Detection, bioMérieux Marcy l’Etoile, France) was used as an additional phenotypic confirmatory test for presumptively detected MRSA strains.

### PCR and SCC*mec* typing

Genomic DNA was extracted from all bacterial cultures and used as a template for amplification.

All isolates were screened for the presence of the *mec*A, *luk*S-PV/*luk*F-PV genes and ACME-locus. PCR experiments were carried out following procedures previously reported [16-18]. The SCC*mec* (Staphylococcal-Chromosomal-Cassette *mec*) types were first determined by a multiplex-PCR protocol previously described, and assigned to the corresponding types [19]. Moreover, for the characterization of the SCC*mec* IV subtype an alternative multiplex-PCR protocol was performed [20], the results were confirmed with other different multiplex-PCR protocols, focusing on the *mec* gene complex and the *ccr* gene complex [21] and *hsd*RMS operon [16].

The sequences of the seven housekeeping genes used for MLST, corresponding to the allelic profile *arc*C-*aro*E-*glp*-*gmk*-*pta*-*tpi*-*yqi*L, were obtained by comparing the sequences obtained for all MRSA strains in study with those in the MLST database http://saureus.mlst.net[22]. All sequence-types (STs) described in the study were deposited on the MLST website. PCR products were analyzed by electrophoresis on 1% or 2% agarose gels (Sigma Aldrich, Saint Louis Mo, USA).

### Statistical methods

Sample size calculation was based on the assumption of a carriage rate of 2%. Around 785 subjects needed to be screened, in order to estimate the carriage rate with adequate precision (95% confidence intervals between 1% and 3% for 785 subjects).

Proportions of the social-demographic characteristics of each group of colonized/not-colonized children were compared through χ^2^ or Fisher exact test where appropriate. Statistical tests were considered significant at a p-value <0.05 level.

In order to study the association between potential risk factors and colonization status at the time of hospital admission, we performed a univariate analysis through a logistic regression model in which odds ratios and 95% CI were calculated. Potential risk factors included in the analysis as independent variables were all those investigated in the questionnaire, plus age at admission.

In each model colonization status was included as a dependent variable, as follows: colonized patients vs non-colonized; MSSA colonized vs non-colonized patients and MRSA colonized vs non-colonized patients. At the univariate level, the analysis was restricted to children aged ≤4 years for “sport”; ≥ 3 years for “nail biting”; <2 years for “breastfeeding”.

Variables with a p-value <0.20 at the univariate analysis were selected to be included in the multivariate analysis using a logistic regression model, with nasal carriage being the dependent variable as reported above. Since none of the variables analyzed on a restricted population at the univariate level had a p value <0.20, the multivariate model included the entire population. Statistical software STATA 11 was used for data analysis.

## Results

### Study population

We enrolled 785 patients from 3 pediatric wards of the Department. Mean age of patients was 4.62 years (median: 2.66, range: 0.03-27.7), of which 403 (51%) were males. Other characteristics of the population are reported in Table 1. Among the enrolled patients, no one had been admitted for a symptomatic *S. aureus* infection or was diagnosed with a nosocomial *S. aureus* infection during hospitalization.

**Table 1:** General characteristics of patients included in the study

		**MSSA**		**MRSA**		**Not colonized**		**Total**	
								**population**	
Number of patients; N		155		9		621		785	
Male; N, %		87	56.1	4	44.4	312	50.2	403	51.3
Age; N, %	0 - 5	95	61.3	8	88.9	459	73.9	562	71.6
	6 - 13	52	33.5	1	11.1	121	19.5	174	22.2
	> = 14	8	5.2	0	0.0	41	6.6	49	6.2
Parent nationality; N, %	Italian	138	89.0	7	77.8	554	89.2	699	89.0
	European	6	3.9	1	11.1	27	4.3	34	4.3
	Extra-European^1^	8	5.2	1	11.1	27	4.3	36	4.6
	Mixed	3	1.9	0	0.0	14	2.3	16	2.0
Mother’s age, years; mean, SD		36.5	7.1	32.9	5.8	35.8	6.7	35.9	6.8
Father’s age, years; mean, SD		38.8	7.3	37.7	6.0	39.0	7.1	38.9	7.1
Mothers with high school diploma; N, %		111	71.6	4	44.4	464	74.7	579	73.8
Fathers with high school diploma; N, %		96	61.9	6	66.7	412	66.3	514	65.5
Unemployed mothers; N, %		62	40.0	5	55.6	230	37.0	297	37.8
Unemployed fathers; N, %		11	7.1	0	0.0	18	2.9	29	3.7
Presence of underlying illness; N, %		58	37.4	4	44.4	160	25.8	222	28.3
Hospitalization length; mean, SD		6.8	6.8	7.6	7.5	7.0	6.0	7	6.2

^1^Among the 36 extra-european patients, 20 (56%) originated from Asian countries, 9 (25%) from African countries, 7 (19%) from South American countries. The only extra-european MRSA colonized patient was from Vietnam.

### Colonization prevalence and risk factors

At admission, a total of 164 patients (20.89%; 95% CI: 18.16%-23.84%) were colonized by *S. aureus*; 155 patients (19.75%; 95% CI: 17.07%-22.64%) were colonized by MSSA and 9 patients (1.15%; 95% CI: 0.5607%-2.093%) were colonized by MRSA.

Among the MRSA colonized patients, 5 had an underlying condition (mucopolysaccharidosis, arthrogryposis, neurofibromatosis, type 1 diabetes, osteoid osteoma). The remaining 4 patients had been admitted with the following diagnoses: bronchiolitis, rickettsiosis, chickenpox, sepsis. Six of the MRSA-colonized patients had already been hospitalized in the last 6 months. Combining information on underlying diseases and previous hospitalizations, 3 out of 9 MRSA colonized patients did not have risk factors for previous healthcare exposures.

Risk factors for MSSA and MRSA carriage identified by multivariate analysis are reported in Table 2.

**Table 2:** **Multivariate risk factors for S. aureus**, **MSSA and MRSA colonization**

	**Risk factor**			**MSSA vs negative**				**MRSA vs negative**			
	**Total N. of patients with risk factor (n. 785)**	**N. of patients MSSA with risk factor (n. 155)**	**N. of MRSA-colonized patients with risk factor (n. 09)**	**OR**	**95% CI**		**p**	**OR**	**95% CI**		**p**
Age 6-13	174	52	1	1,77	1,04	2,98	0,034	NS			
Unemployed father	29	11	0	2,67	1,19	5,99	0,017	NS			
Chronic illness	222	58	4	1,56	1,01	2,39	0,043	NS			
Antibiotics last 6 months	509	92	4	0,65	0,45	0,97	0,034	0,66	0,46	0,96	0,033
Hospital admission last 6 months	319	63	6	NS				4,92	0,97	24,83	0,053

*OR* = Odds Ratios; *CI* = Confidence Intervals.

According to the multivariate analysis, compared to non-colonized patients, MRSA colonized patients were more likely to have been hospitalized in the previous 6 months. Antibiotic therapy in the last 6 months was found as a protective factor for MRSA colonization. The multivariate analysis did not identify age as a risk factor for MRSA colonization, while MSSA-colonized patients were more likely to belong to the 6–13 age group compared to non-colonized children.

### MRSA antimicrobial susceptibility and molecular characteristics

Antimicrobial susceptibility and molecular characteristics of the isolates are reported in Table 3.

**Table 3:** **Patient**’**s diagnosis**, **molecular characteristics and antibiotic resistance of MRSA colonizing isolates**

	**Diagnosis**	**ST**	**SCC*mec***	***mec***-**complex**	***ccr***-**complex**	**PVL**	**OXA**	**E**	**DA**	**CN**	**CIP**	**RD**	**SXT**	**VA**	**TEC**	**LNZ**	**MUP**
1	Mucopolysaccharidosis	8	IV.c	B	A2B2	-	R	R	S	S	R	R	R	0,5	0,25	2	1
2	Chickenpox	8	IV.a	B	A2B2	-	R	S	S	S	R	S	S	1	0,25	2	0,25
3	Bronchiolitis	8	IV.a	B	A2B2	+	R	S	S	S	S	S	S	0,5	0,5	2	1
4	Arthrogryposis	22	IV.c	B	A2B2	-	R	S	S	S	S	S	S	1	0,25	2	0,5
5	Neurofibromatosis 1	45	IV.a	B	A2B2	-	R	R	S	S	S	R	S	0,5	1	2	4
6	Type 1 diabetes	15	IV.a	B	neg	-	R	S	S	S	R	S	S	0,5	0,25	2	2
7	Osteoid osteoma	5	IV.a	B	A2B2	-	R	R	S	S	S	S	S	1	0,25	4	0,25
8	Rickettsiosis	1	IV.a	B	A2B2	-	R	R	S	R	S	R	S	0,5	0,25	2	1
9	Sepsis	30	V	C	C	-	R	S	S	S	S	S	S	0,5	0,25	2	2

*OXA*, oxacillin; *E*, erythromycin; *DA*, clindamycin; *CN*, gentamicin; *CIP*, ciprofloxacin; *RD*, rifampicin; *SXT*, trimethoprim-sulfamethoxazole; *VA*, vancomycin; *TEC*, teicoplanin; *LNZ*, linezolid; *MUP*, mupirocin. *R* = resistant; *S* = sensitive.

Nine MRSA were isolated, all characterized by phenotypic and molecular methods, as described in the Methods section.

All isolates were oxacillin resistant, 4/9 were erythromycin resistant, 3/9 ciprofloxacin and rifampicin resistant, 1/9 gentamicin resistant, and only one isolate was resistant to cotrimoxazole. All strains were susceptible to clindamycin, mupirocin, linezolid and glycopeptides.

MLST analysis showed 7 different STs associated with different variants of SCC*mec* IV and only one with SCC*mec* V.

Only 1 strain carried *lukS*-*PV*/*lukF*-*PV* genes, and did not harbour the arginine catabolic mobile element (ACME), associated toUSA300 clone.

## Discussion

This study assessed MRSA nasal colonization in a population of children admitted to a General Pediatric Department, including a large group of patients affected by chronic diseases. Our results show an MSSA nasal colonization prevalence of 19.75% and an MRSA nasal colonization prevalence of 1.15%.

MRSA nasal colonization in the pediatric age has been investigated by a number of studies, which showed variable prevalence figures and diverse risk factors [7,8,23-27]. Most studies investigated carriage in a healthy pediatric population [7,25,27]. A smaller number of studies focused on Intensive Care Units [28,29], or on patients with underlying diseases [24]. Few studies investigated colonization in a general pediatric ward and our study gives new insights on this topic. Two studies [30,31] investigated MRSA nasal carriage in different hospital departments, including a pediatric ward, and in both studies no MRSA was identified. In a multicenter study carried out in children’s hospitals in Switzerland, one MRSA was identified in 1,350 investigated patients (prevalence: 0.07%) [8].

We molecularly characterized the 9 MRSA isolates and demonstrated the presence of different strains with community or nosocomial origins.

A circulation of the ST8-CAMRSA-IV.a clone in Italy has been already demonstrated in patients affected by invasive infections (necrotizing pneumonia, skin and soft tissue infections, sepsis), always in association with PVL production. Among the colonizing isolates belonging to the ST8-CAMRSA-IV.a clone, only one carried the *lukS*-*PV*/*lukF*-*PV* genes [32].

The ST1-CAMRSA-IV.a, already described as USA400 in the United States, circulates worldwide in different countries [33], and in Italy was recently isolated from pigs (nasal samples and dust samples collected in holdings of breeding and production pigs) [34].

The ST30 clones, with different pulsotype and genetic characteristics, have been reported in many areas of the world. This clone was previously described in Italy in a patient with necrotizing pneumonia, and associated with SCC*mec* IV and the *lukS*-*PV*/*lukF*-*PV* genes [32]. On the contrary, our isolate carried for the first time SCC*mec* V and *ccr*C type recombinases, and no *lukS*-*PV*/*lukF*-*P* genes. This variability in acquiring diverse genetic elements (SCC*mec* and PVL-encoding phages) suggested that this clone, in Italy, has evolved in multiple occasions.

Circulation of the ST45-CAMRSA-IV.a has been recently demonstrated in Cambodian children [35]. Interestingly, our ST45-CAMRSA-IV.a was isolated from a recently adopted Vietnamese child.

ST22-CAMRSA-IV was previously described as an epidemic infective clone in adults (EMRSA 15) [36] and has been recently isolated on Portuguese public buses [37]. *S. aureus* belonging to ST15 have rarely been reported as methicillin-resistant. One colonizing ST15-MRSA was reported from Africa and ST15 was previously associated with community-onset MSSA bacteremia among adults in an Italian study [38,39]. The present study identified one of the MRSA colonizing isolates showing ST15-SCC*mec* type IV.

The only ST5 SCC*mec* IV colonized a child with previous hospitalizations for an osteid osteoma.

We observed a tendency to statistical significance in the association between MRSA colonization and hospitalization in the 6 months prior to the nasal sampling, confirming the results reported by other authors [40,41]. Nevertheless, the majority of our MRSA isolates have community origins. This observation shows that strains that circulate in the community also circulate in hospital settings. To this regard, more research is needed to investigate the role of children that have frequent contacts with health care institution (eg. children affected with underlying disease and chronic conditions) as potential MRSA vectors between the hospital and the community [6].

According to our findings, use of antibiotics in the 6 months before admission protects from both MSSA and MRSA carriage. Other authors have previously reported a protective effect of antibiotic therapy in the last 6 months on MSSA colonization [25,26].

In the complex interaction of factors affecting emergence and spread of multridrug-resistant organisms, antimicrobials are known to exert a selective pressure on resistant clones. We found, instead, a protective effect of antibiotics administered in the previous 6 months on MRSA colonization. In order to elucidate this result, more data regarding prescriptions (kind of infection, class of prescribed antibiotics, days of therapy) would be needed. Although, this kind of information is easily subject to recall bias, therefore we decided not to include it in our questionnaire. A cohort study with a prospective design would be needed for appropriately characterizing the effect of antibiotic use on resistant clones.

Our study has some limitations. First, parents not speaking Italian, English, Spanish and French were not enrolled in the study. This might have led to an underestimation of the MRSA carriage prevalence. However, as foreign patients are a very limited proportion of the total hospitalized patients, the effect of this selection bias could be negligible.

We did not explore colonization in other body sites (throat, perineum), which could have led to a misclassification of colonized patients as non-colonized [42]. Another possible limitation is the low number of patients that were positive to MRSA for which risk factors were evaluated. Thus, associations found in our study may be not sufficient to explain the outcome (nasal carriage).

## Conclusion

Our findings show that strains that circulate in the community also circulate in hospital settings: in fact, the majority of our MRSA colonizing isolates have community origins; nevertheless, most MRSA-colonized patients had been hospitalized previously. More research is needed to elucidate the role of antibiotic prescriptions in the emergence of MRSA clones, and to analyze the possible role of children with frequent contact with health care centers (eg. children affected with chronic diseases) as MRSA vectors between the hospital and the community.

## Competing interests

The authors declare that they have no competing interests.

## Authors’ contributions

FG coordinated the study and drafted the manuscript, MO and LM performed the microbiological analyses, DB and FC performed the molecular genetic analyses, EC performed the statistical analysis, DV coordinated patient enrollment, CR and AV participated in drafting the manuscript, MR revised the manuscript, AET and SS supervised the study, revised the manuscript and gave final approval of the version to be published. All authors read and approved the final manuscript.
